# The role of RNA m^6^A methylation in the regulation of postnatal hypoxia-induced pulmonary hypertension

**DOI:** 10.1186/s12931-021-01728-6

**Published:** 2021-04-26

**Authors:** Shanshan Xu, Xuefeng Xu, Ziming Zhang, Lingling Yan, Liyan Zhang, Lizhong Du

**Affiliations:** 1 Department of Neonatology, The Children’s Hospital, Zhejiang University School of Medicine, Hangzhou, 310052 People’s Republic of China; 2 Department of Rheumatology, The Children’s Hospital, Zhejiang University School of Medicine, Hangzhou, 310052 People’s Republic of China; 3 Fuzhou Children Hospital of Fujian Medical University, Fuzhou, 350005 People’s Republic of China

**Keywords:** Pulmonary hypertension, Postnatal hypoxia, N^6^-methyladenosine, RNA methylation

## Abstract

**Background:**

Pulmonary hypertension (PH) is a complex pulmonary vascular disease characterized by an imbalance in vasoconstrictor/vasodilator signaling within the pulmonary vasculature. Recent evidence suggests that exposure to hypoxia early in life can cause alterations in the pulmonary vasculature and lead to the development of PH. However, the long-term impact of postnatal hypoxia on lung development and pulmonary function remains unknown. N^6^-methyladenosine (m^6^A) regulates gene expression and governs many important biological processes. However, the function of m^6^A in the development of PH remains poorly characterized. Thus, the purpose of this investigation was to test the two-fold hypothesis that (1) postnatal exposure to hypoxia would alter lung development leading to PH in adult rats, and (2) m^6^A modification would change in rats exposed to hypoxia, suggesting it plays a role in the development of PH.

**Methods:**

Twenty-four male Sprague–Dawley rats were exposed to a hypoxic environment (F_i_O_2_: 12%) within 24 h after birth for 2 weeks. PH was defined as an increased right ventricular pressure (RVP) and pathologic changes of pulmonary vasculature measured by α-SMA immunohistochemical staining. Methylated RNA immunoprecipitation sequencing (MeRIP-seq) was performed to analyze m^6^A modification changes in lung tissue in 2- and 9-week-old rats that were exposed to postnatal hypoxia.

**Results:**

Mean pulmonary arterial pressure, lung/body weight ratio, and the Fulton index was significantly greater in rats exposed to hypoxia when compared to control and the difference persisted into adulthood. m^6^A methyltransferase and demethylase proteins were significantly downregulated in postnatal hypoxia-induced PH. Distinct m^6^A modification peak-related genes differed between the two groups, and these genes were associated with lung development.

**Conclusions:**

Our results indicate postnatal hypoxia can cause PH, which can persist into adulthood. The development and persistence of PH may be because of the continuous low expression of methyltransferase like 3 affecting the m^6^A level of PH-related genes. Our findings provide new insights into the impact of postnatal hypoxia and the role of m^6^A in the development of pulmonary vascular pathophysiology.

## Introduction

Pulmonary hypertension (PH) is a complex pathology regulated by a multitude of molecular pathways and processes. Evidence suggests that postnatal environmental factors can negatively impact the development and physiological status of the pulmonary vasculature [1–3]. These alterations in pulmonary maturation and function could result in the development of PH in adulthood, increase morbidity and mortality, and significantly reduce the quality of life of this population. Previous results from our laboratory revealed that embryonic growth is related to PH and pulmonary vascular function, as intrauterine growth retardation (IUGR) resulted in decreased pulmonary vascular growth [1] and extrauterine growth retardation (EUGR) was associated with varying degrees of pulmonary arterial hypertension (PAH) later in life [1, 3]. These results suggest that environmental factors in early life play a critical role in developing PH and result in long-term ramifications for pulmonary function.

As part of the epigenetic regulation in mammals, RNA modifications, similar to DNA and histone modifications, play an essential role in various physiological and pathological processes [4, 5]. More than 100 distinct modifications of RNA can occur [6], with N^6^-methyladenosine (m^6^A) being the most abundant and prevalent chemical modification. This dynamic and reversible modification is regulated by the methyltransferase (methyltransferase like 3 (METTL3), methyltransferase like 14 (METTL14), and wilms’ tumor 1-associating protein (WTAP), called “writers”), demethylase (fat mass- and obesity- associated protein (FTO) and a-ketoglutarate-dependent dioxygenase alkB homolog 5 (ALKBH5), called “erasers”), and RNA-binding proteins (e.g., the YTH domain-containing family (YTH family), called “readers”) [7, 8]. Several investigations have demonstrated that m^6^A regulates mRNA stability [9], translation [10], nuclear export [11], and decay [12] and participates in many biological processes [13–16]. Of note, m^6^A levels change during organ maturation and early life processes [17–19], and several investigations have suggested a temporal progression of m^6^A modification of different tissues and different stages of life [20, 21]. Despite these prospects, the dynamic m^6^A changes in lung or pulmonary vascular diseases remain to be elucidated.

In addition to hypoxia-induced changes in pulmonary function, several investigations have shown that epigenetic modifications, including DNA methylation and histone modification, play a vital role in the development of pulmonary diseases [22, 23]. While many investigations have provided insight into the epigenetic mechanisms of PH, few have focused on RNA modifications and their impacts on the development of this disease [24]. Recent investigations suggest that environmental factors in early life could predispose individuals to PH via epigenetic mechanisms [25]. However, whether RNA modifications are associated with the development of PH following postnatal exposure to hypoxia remains unknown.

Considering the essential role of m^6^A in tissue structure and function throughout life and the potential for postnatal exposure to hypoxia to alter lung development, the purpose of this investigation was to assess the impact of postnatal exposure to hypoxia on long-term lung maturation, the development of PH, and the role of m^6^A in these changes. We tested the two-fold hypothesis that (1) postnatal exposure to hypoxia would alter lung development leading to PH in adult rats and (2) m^6^A modifications would be altered in rats exposed to hypoxia. Using a postnatal hypoxia rat model, we assessed pulmonary arterial pressure and evaluated pulmonary vasculature function. We also performed methylated RNA immunoprecipitation sequencing (MeRIP-seq) to confirm the potential role of m^6^A in pulmonary vascular function following exposure to hypoxia.

## Methods

### Ethical approval and animal care

All procedures outlined herein followed the guidelines established by the National Institutes of Health of China. All animal procedures were reviewed and approved by the Institutional Animal Care and Use Committee of Zhejiang University (ZJU20160215).

Sprague–Dawley rats were purchased from the Slaccas Company (Shanghai, China) and bred in our laboratory. The hypoxia model was established in a hypoxia chamber (Biospherix, USA), where pups were exposed to hypoxia together with their dams. Each dam was kept in an individual cage with her pups and she had free access to water and food. The whole cage was placed into the hypoxia chamber. The pups assigned to the hypoxia group (n = 24) were exposed to hypoxia (F_i_O_2_ 11–12%) within 24 h after birth for 2 weeks before being returned to normoxic conditions (four pups died during hypoxia). The control group (n = 16) lived under normoxic conditions throughout their life. Eight control and 12 hypoxia animals were sacrificed at 2 weeks; another 8 control and 8 hypoxia animals were sacrificed at 9 weeks. From each group, 5 animals’ left lung were used for histological analysis, 6–8 animals’ right lung were used to extract RNA and protein for qPCR, m^6^A level detecting and western blot, and 3 animals were used for RNA sequencing. All animals were sacrificed using sodium pentobarbital anesthesia at either 2 weeks or 9 weeks old. Normal saline (10–20 ml) was injected from the pulmonary artery to reduce the effect of red blood cells. The right lung tissue was removed and then transferred to liquid nitrogen immediately. The left lung was fixed in 10% formalin for at least 48 h before being cut for slides.

### Pulmonary arterial pressure assessment

Prior to euthanasia, right ventricular pressure (RVP) was measured in 2-week-old rats and mean pulmonary arterial pressure (mPAP) in 9-week-old rats as described previously [26]. Briefly, 2% pentobarbital sodium (50 mg/kg, intraperitoneally) was used to anesthetize rats. They were then placed on a surgical table. In 2-week-old anesthetized rats, tracheal intubation was performed, and the thorax was opened. A catheter was then inserted into the right ventricle to measure the right ventricular pressure. For the measurement of mPAP (9 weeks), a polyethylene catheter (PE-50) was inserted into the pulmonary artery via the jugular vein and the right heart to measure pulmonary arterial pressures. RVP or mPAP was recorded when the waveform was stable. The Fulton index [RV / (LV + S)] was also used to determine the degree of cardiac remodeling, where RV represents the weight of the right ventricle and (LV + S) represents the weight of the left ventricle plus septum.

### Hematoxylin–eosin staining (HE) and Immunohistochemical staining (IHC)

Pulmonary pathological changes were analyzed by HE and IHC, as described in detail previously [3]. The paraffin blocks were cut into sections of 5 µm thickness. The primary antibodies used in IHC were anti-α-SMA (1:500, Servicebio, China). All image data were processed using Image-Pro Plus software. Microphotographs of HE-stained sections were taken under a 200 × microscope and those without trachea or large blood vessels were selected at random from each HE-stained section. The area (A) of the image and number of alveoli (Na) were then calculated in each image. The mean alveolar number (MAN) was Na/A (pieces/cm^2^). MAN reflects alveolar density [27]. Microphotographs of IHC-stained sections were taken under a 200 × microscope and 400 × microscope. We used microphotographs under a 400 × microscope for data analysis. The area of the pulmonary vascular adventitia, the area of the pulmonary vascular intima, and the outer perimeter of pulmonary vascular basement membrane (Pbm) were then calculated. The area of the pulmonary vascular intima was subtracted from the area of the pulmonary vascular adventitia to obtain the α-SMA-stained area. The α-SMA-stained area/Pbm reflects the pulmonary vascular muscularization [28]. For MAN, at least 6 fields of view per animal were analyzed. For pulmonary arterial medial wall thickness assessment, 5–10 pulmonary arteries per animal were analyzed, and arteries with a diameter between 50 and 150 µm were selected.

### Pulmonary vascular endothelial cell (PVEC) isolation

Magnetic activated cell sorting (MACS) was used to separate PVECs from lung tissue. The required reagents and consumables were purchased from Miltenyi (Germany), and the extraction steps were performed according to the manufacturer’s instructions. Separated cells were frozen at − 80 °C for further use.

### Quantitative real-time PCR (qPCR)

Lung tissue total RNA was isolated using a Multisource RNA Miniprep kit (Axygen, USA). The total RNA was reverse transcribed into cDNA using PrimeScript™ RT Master Mix (Perfect Real Time) Kit (Takara, China). qPCR was performed using an ABI Prism 7500 instrument following the SYBR-Green reagent protocol (Takara, China). Data were analyzed as fold change by the 2^−ΔΔCt^ method. Primers used in this study are listed below: β-actin forward: 5′-gccaaccgtgaaaagatg-3′, β-actin reverse: 5′-tgccagtggtacgaccag-3′; Rras2 forward: 5′-gaccatggcttttgcttgct-3′, Rras2 reverse: 5′-tagcggggacattgaacgtg-3′; Ttll12 forward: 5′-gcatccagagagttcgcaga-3′, Ttll12 reverse: 5′-gggtctcgggtgtaacacag-3′; Nog forward: 5′-tgtacgcgtggaacgaccta-3′, Nog reverse: 5′-ggcttacacaccatgccctc-3′; Vangl2 forward: 5′-tgatccccgattgcttggtc-3′, Vangl2 reverse: 5′-ccagaccactcggctgttt-3′; Gli3 forward: 5′-atcagccctgctttgagctt-3′, Gli3 reverse: 5′-gatgggtctctgcgttggaa-3′; Trps1 forward: 5′-gagcagcagaggatctggag-3′, Trps1 reverse: 5′-cctagtctgctccccgtttg-3′; Tc1 forward: 5′- ctcttcgagtaagcccgtcc-3′, Tc1 reverse: 5′-gggctcgagttttctcctcc-3’.

### Protein and western blot

Lung tissue was lysed in a RIPA buffer (Beyotime Biotechnology, China) with PMSF (Thermo Fisher, USA); and a phosphatase inhibitor cocktail (Thermo Fisher, USA) was also added to the RIPA buffer when p-eNOS was to be analyzed. The lysate was then centrifuged for 10 min at 12,000*g*, and the supernatant was collected. An enhanced BCA protein assay kit (Beyotime Biotechnology, China) was used to measure the protein concentration. A 10% SDS-PAGE gel was used for electrophoresis, and the electrotransfer occurred for 90 min at 110 V with 0.45 µm PVDF membranes (Merck Millipore, USA). The membranes were incubated with antibodies, and a chemiluminescence method was used for color development. The G:BOX system (Syngene, USA) was used to generate gray-scale images. Image-Pro Plus software (Media Cybernetics, USA) was used to quantify the amount of protein as gray value. The primary antibodies used in the western blots were as follows: anti-METTL3 (1:1000, ab195352, Abcam, UK), anti-METTL14 (1:2000, #51104, Cell Signaling Technology, USA), anti-WTAP (1:1000, #56501, Cell Signaling Technology), anti-FTO (1:1000, ab92821,Abcam), anti-ALKBH5 (1:500, 16837–1-AP,Proteintech, USA), anti-VEGF (1:200, 26157–1-AP,Proteintech), anti-eNOS (1:1000, ab199956,Cell Signaling Technology), anti-p-eNOS (1:1000, #9570, Cell Signaling Technology), and anti-β-actin (1:5000, A1978, Sigma-Aldrich, USA).

### Total RNA m^6^A level detection

We used an EpiQuik™ m^6^A RNA Methylation Quantification Kit (Colorimetric) (Epigentek, USA) to determine the total m^6^A level in the lung tissue. The main procedure included three steps: RNA combination, m^6^A RNA capturing, and absorbance detection. The total m^6^A level was calculated using formulas provided in the kit.

### Methylated RNA immunoprecipitation sequencing (MeRIP-seq)

MeRIP was based on the m^6^A-seq protocol [29]. In brief, RNA samples were purified and then fragmented into 100 nt through chemical fragmentation. We then used anti-m^6^A antibodies to immunocapture m^6^A-modified RNA fragments. After elution with free m^6^A, library preparation and massively parallel sequencing was performed.

### MeRIP data analysis

Distributions of peaks and motifs were analyzed using MACS2 and HOMER software. Gene Ontology (GO) analysis and Kyoto Encyclopedia of Genes and Genomes (KEGG) pathway enrichment analysis were applied to analyze differentially expressed genes (DEGs) related to m^6^A modification. GO analysis was used to classify DEG functions into biological process, molecular function, and cellular component. KEGG analysis was performed to identify the pathways the DEGs were enriched in.

### Statistical analysis

All results were obtained from at least three biological replicates and shown as means ± standard error of the mean (means ± SEM). GraphPad Prism 6.0 and SPSS 23.0 were used for statistical analyses. The Student’s *t*-test was used to analyze the difference between independent samples. Comparisons were considered statistically significant when the *P-*value was < 0.05.

## Results

### Postnatal hypoxia dysregulates lung development and leads to PH

**Fig. 1:**
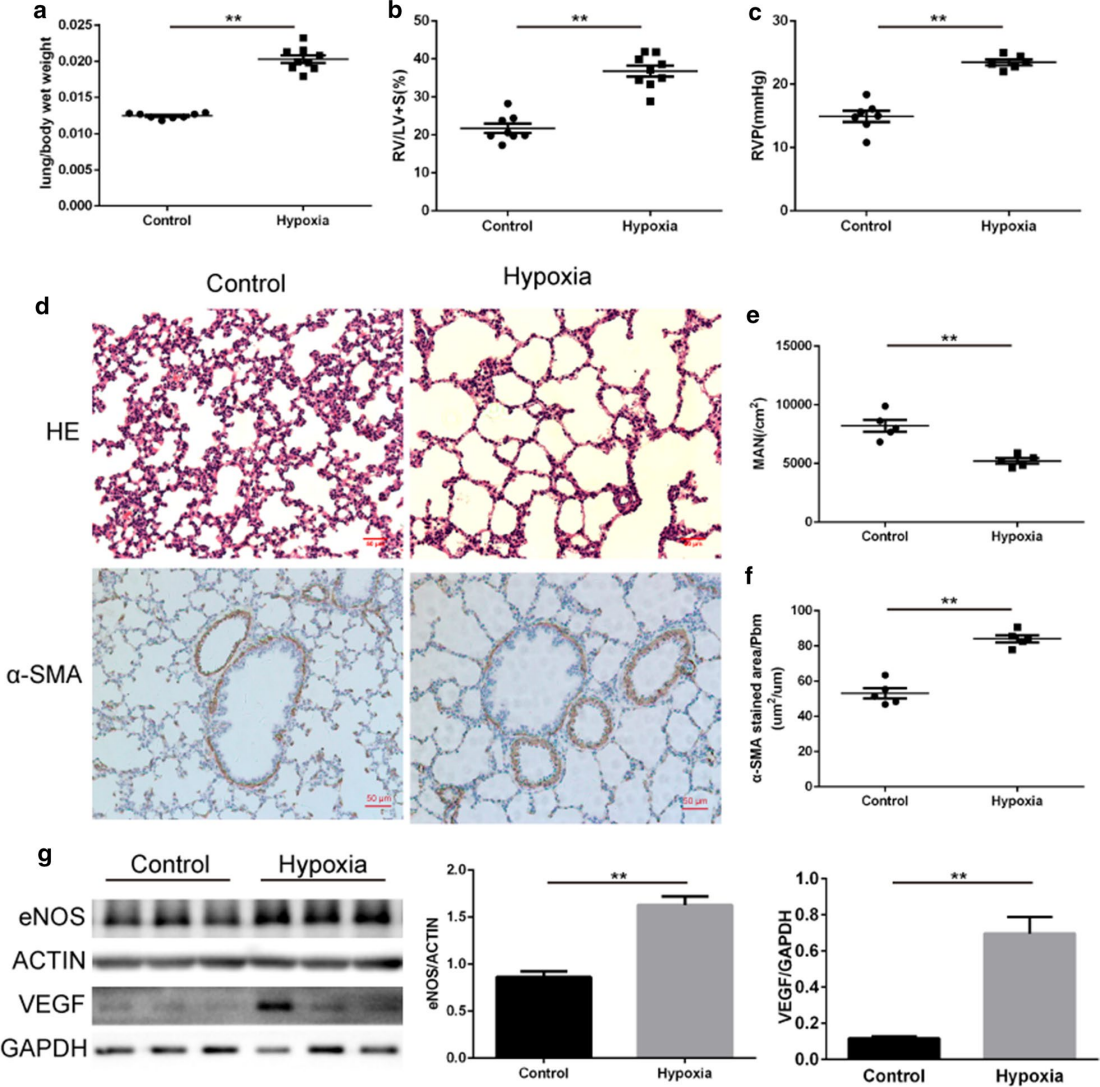
Postnatal hypoxia resulted in PH. **a** Comparison of lung/body wet weight ratio after 2 weeks of hypoxia (N = 8–9 per group). **b** Right ventricular hypertrophy index [RV/(LV + S)] differed between hypoxia and normoxia rats (N = 8–9 per group). **c** Comparison of right ventricular pressure (RVP) in the two groups (N = 6–7 per group). **d** HE staining and immunohistochemical staining of lung tissue (N = 5 per group). **e** Mean alveolar number (MAN) of the two groups (N = 5 per group). **f** The thickness of pulmonary arterial intima measured by α-SMA (N = 5 per group). **g** Expression of eNOS and VEGF in the lung (N = 6 per group). *P < 0.05, **P < 0.01 compared to the control group

Lung/body wet weight ratio increased after 2 weeks of exposure to hypoxia (hypoxia vs. control groups: 2.03 ± 0.05% vs. 1.25 ± 0.01%), suggesting that pulmonary edema occurred in the hypoxia group. RVP (23.47 ± 0.44 mmHg vs. 14.91 ± 0.88 mmHg) and RV/(LV + S) (36.78 ± 1.44% vs. 21.73 ± 1.24%) were both significantly increased, suggesting that postnatal hypoxia caused PH and the associated remodeling of cardiac tissue (Fig. 1a–c). The MAN suggested that the development of alveoli in the hypoxia group was delayed when compared to controls (Fig. 1d,e). α-SMA immunostaining indicated that the pulmonary arterial intima was thickened in the hypoxia group (Fig. 1d,f). We also assessed the expression of endothelial nitric oxide synthase (eNOS) and vascular endothelial growth factor (VEGF), which play essential roles in the development of PH (Fig. 1g). The two proteins were both highly expressed in the lung tissue of the hypoxia group. Collectively, postnatal hypoxia of newborn rats dysregulated lung development and led to PH.

### Postnatal hypoxia decreased the expression of m^6^A related proteins

**Fig. 2:**
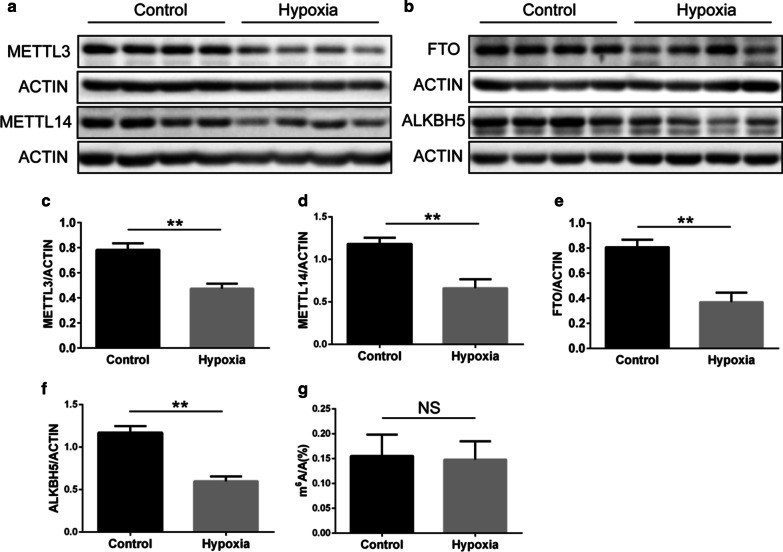
The expression of m^6^A-related proteins after postnatal hypoxia. **a**, **b** Western blot images showing the expression of m^6^A methyltransferase and demethylase proteins (N = 6). **c**–**f**. Quantitative analysis of the western blot experiments (N = 6 per group). **g** The total RNA m^6^A level in the lung tissue (N = 5–8 per group). *P < 0.05, **P < 0.01 compared to the control group

To further explore m^6^A methylation after exposure to postnatal hypoxia, we determined the expression of five m^6^A-related proteins in lung tissue. A decrease in the m^6^A methyltransferases METTL3 and METTL14 was found (Fig. 2a,c,d). The demethylase proteins FTO and ALKBH5 were also decreased following postnatal hypoxia (Fig. 2b, e, f), but the expression of WTAP was not different between groups (data not shown). There was no clear impact of postnatal hypoxia on total m^6^A in the lung tissue (Fig. 2g).

### Postnatal hypoxia changed the m^6^A methylation in lung tissue

**Fig. 3:**
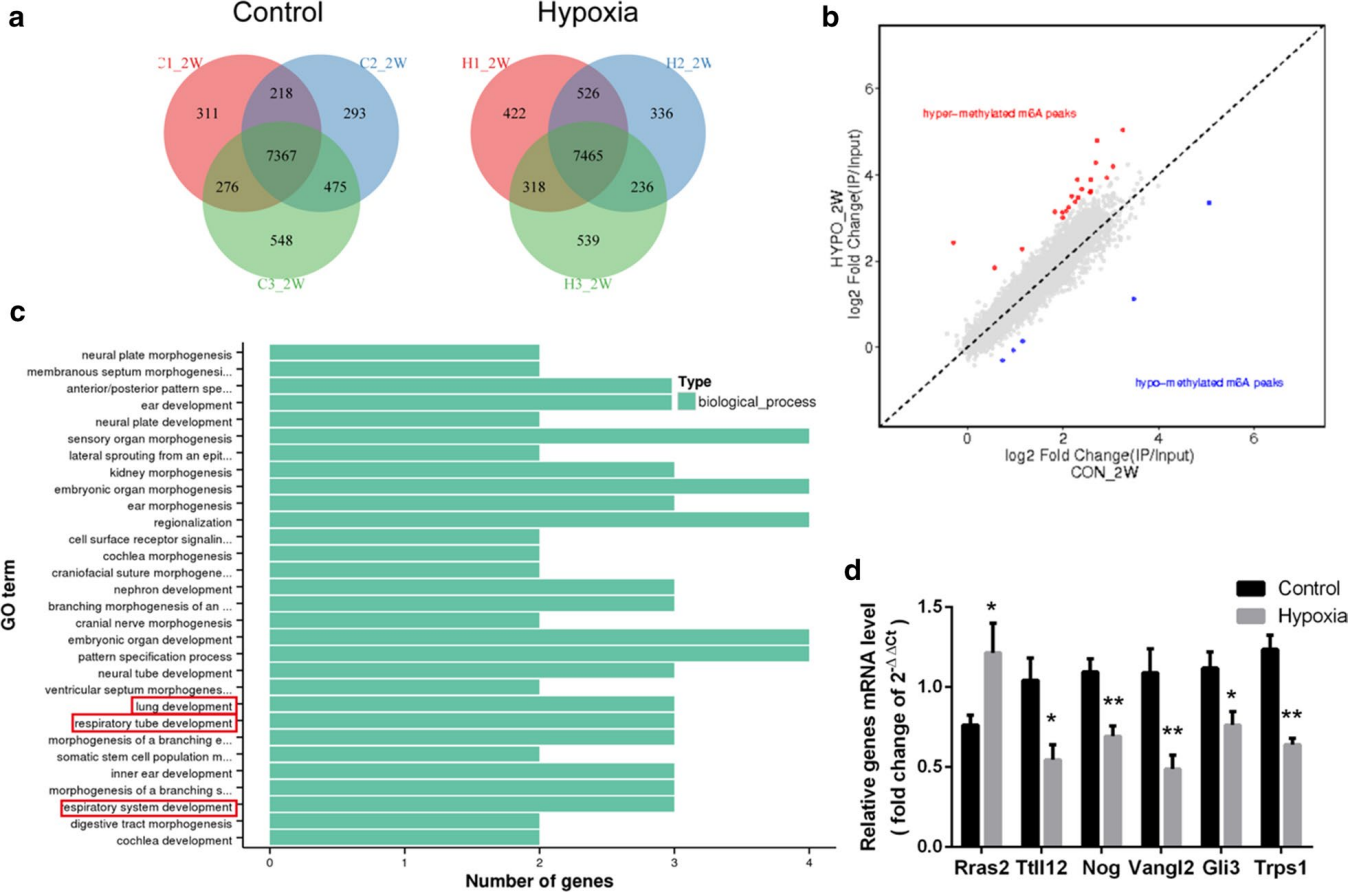
m^6^A methylation after 2 weeks hypoxia. **a** Numbers of m^6^A peaks shown as a Venn diagram (N = 3 per group). **b** Distinct m^6^A peaks by peak comparison between the two groups (N = 3 per group). **c** GO analysis of the differential methylated peaks after hypoxia (N = 3 per group). **d** mRNA levels of differential peak-related genes (N = 6 per group). *P < 0.05, **P < 0.01 compared to the control group

MeRIP was then utilized to assess the specific m^6^A methylation changes following hypoxia. We identified 9488 methylated poly(A) peaks in the control group and 9842 in the hypoxia group (Fig. 3a). By comparing the m^6^A methylation peaks in the two groups, we found that 21 peaks were hyper-methylated, and 5 peaks were hypo-methylated in the hypoxia group (Fig. 3b). Then we performed GO analysis to identify the possible functions of the genes related to these differential peaks. As shown in Fig. 3c, these genes acted in many biological processes associated with organ morphogenesis and development. Data showed three of them- Nog, Vangl2 and Gli3 participated in lung development, respiratory tube development, and respiratory system development.

Further verification was done by qPCR as we tested five differential peak-related genes (Rras2, Ttll12, Ccr3, Ccr6 and Trps1) with known contributions to lung diseases. mRNA levels from three genes (Rras2, Ttll12 and Trps1) changed in coordination with their m^6^A modification (Fig. 3d). Also, mRNA levels of Nog, Vangl2 and Gli3 were decreased after postnatal hypoxia (Fig. 3d). In summary, m^6^A methylation participates in the regulation of vascular-related gene expression following postnatal hypoxia.

### The long-term effect of postnatal hypoxia on pulmonary vascular in adult rat

**Fig. 4:**
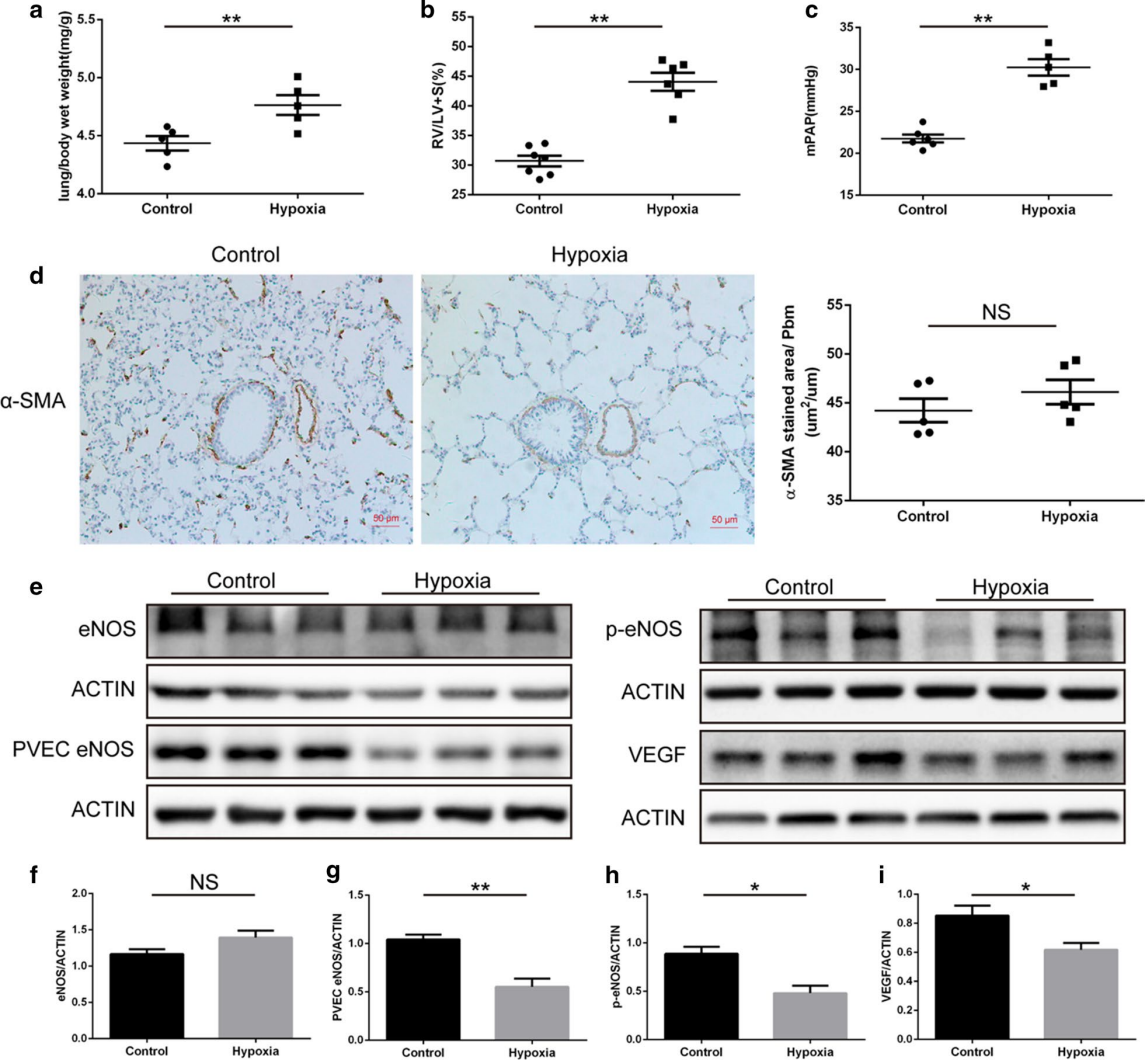
The long-term effect of postnatal hypoxia on the pulmonary vasculature of adult rats. **a** A comparison of lung/body wet weight ratio in adult rats who had been exposed to postnatal hypoxia (N = 6 per group). **b** Right ventricular hypertrophy (Fulton) index [RV / (LV + S)] differed between hypoxia and normoxia rats at 9 weeks old (N = 6–7 per group). **c** Comparison of mean pulmonary arterial pressure (mPAP) between the groups (N = 5–6 per group). **d** Immunohistochemical staining of lung tissue and the thickness of pulmonary arterial intima measured by α-SMA (N = 5 per group). **e** Western blot images of PH-related proteins in the two groups (N = 6 per group). **f**–**i** Quantitative analysis of the Western blot experiments (N = 6 per group). *P < 0.05, ** *P* < 0.01 compared to the control group

To assess the influence of postnatal hypoxia on pulmonary function in adulthood, we measured the lung/body wet weight ratio, RV/(LV + S), and mPAP in 9-week-old rats. We found that the lung/body wet weight ratio, RV/(LV + S), and mPAP were significantly higher in the hypoxia group than in the controls (Fig. 4a–c). α-SMA immunostaining indicated that the thickness of pulmonary arterial intima had no difference between the two groups (Fig. 4d). These results suggest that postnatal hypoxia-induced PH could continue to adulthood and the proliferation of pulmonary vascular smooth muscle cells may not be the main reason for the increase in pulmonary artery pressure over long durations.

Western blot was used to assess eNOS and VEGF protein levels. VEGF and phosphorylated (p-eNOS) were lower in the hypoxia group than in the controls (Fig. 4e,h,i), although there was no difference in eNOS protein expression between the two groups (Fig. 4e,f). Also, the expression of eNOS in PVECs was lower in the hypoxia group than in the controls (Fig. 4e,g). These results suggest that pulmonary vasculature dysfunction perpetuates in adult rats that have experienced postnatal hypoxia.

### The long-term effect of postnatal hypoxia on m^6^A methylation in the adult rat

**Fig. 5:**
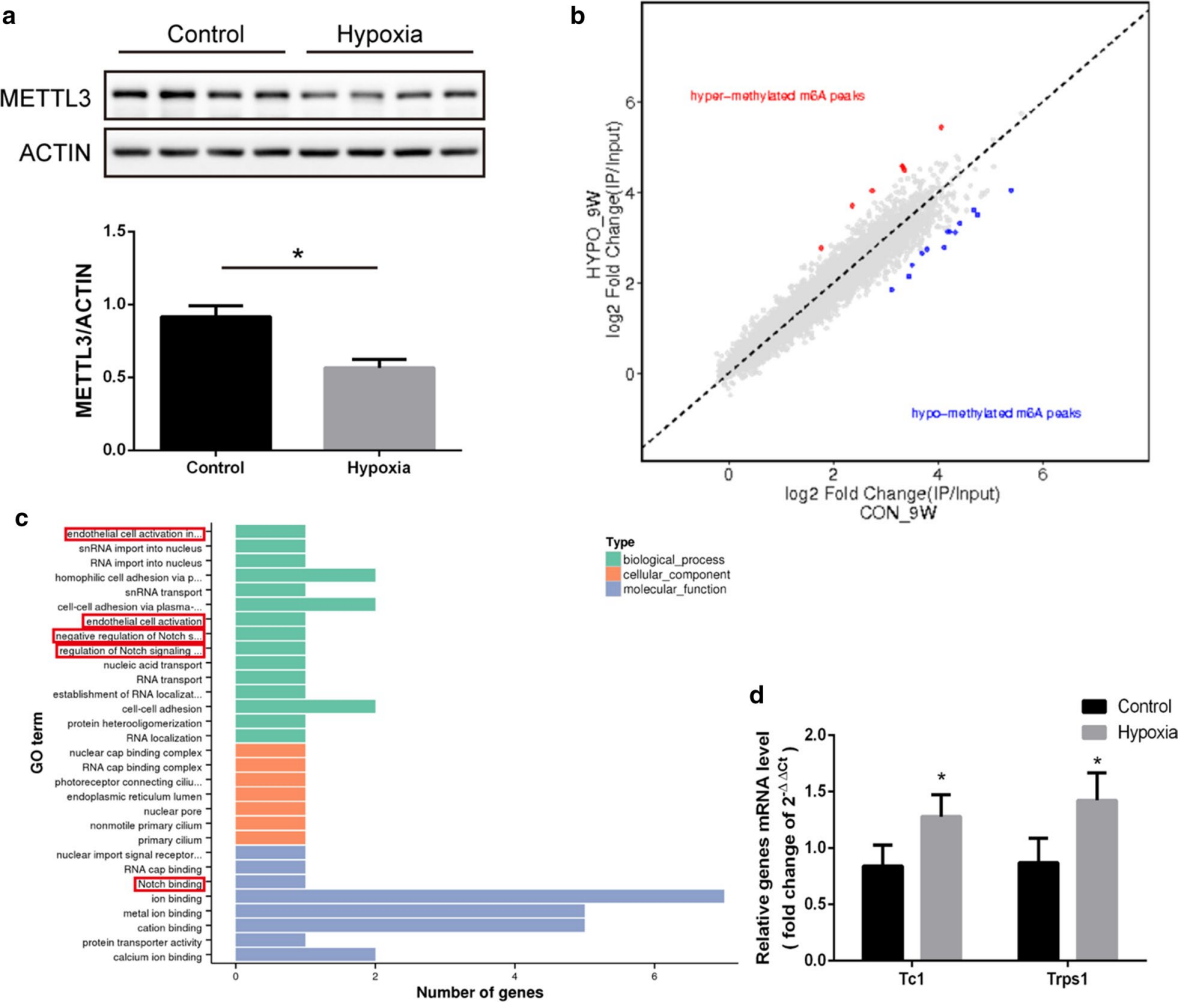
m^6^A methylation following postnatal hypoxia in 9-week-old rats. **a** Expression of METTL3 in adult rats (N = 6 per group). **b** Distinct m^6^A peaks by peak comparison between the two groups (N = 3 per group). **c** GO analysis of the differential methylated peaks in adult rat after postnatal hypoxia (N = 3 per group). **d** mRNA levels of differential peak-related genes in 9-week-old rats (N = 6 per group). *P < 0.05, **P < 0.01 compared to the control group

The expression of five m^6^A-related proteins during adulthood was measured in lung tissue from both groups. METTL3 was lower in the hypoxia group than in the controls (Fig. 5a), but there were no differences in the other four m^6^A-related proteins between the groups (data not shown).

Next, we performed MeRIP again to find the between-group specific m^6^A methylation changes. Although there were only 7 peaks hyper-methylated and 13 peaks hypo-methylated in the adult rats who were exposed to postnatal hypoxia (Fig. 5b), GO analysis showed that Tc1 was involved in the regulation of Notch signal pathways and the activation of endothelial cells (Fig. 5c). We also did qPCR to verify mRNA level, data showed mRNA levels of Tc1 and Trps1 were increased in adult rat after postnatal hypoxia (Fig. 5d).

## Discussion

The present investigation explored the acute and long-term impacts of postnatal hypoxia on pulmonary vascular function and remodeling and the role of m^6^A in the development of PH. Consistent with our original hypothesis, postnatal exposure to hypoxia resulted in significant remodeling of the pulmonary vasculature, increased mPAP, and reduced levels of m^6^A-related proteins. The reduced METTL3 expression was sustained into adulthood. These results suggest that m^6^A modification might be involved in the PH pathological process initiated by postnatal exposure to hypoxia.

Based on many epidemiological reports, the Developmental Origins of Health and Disease (DOHaD) theory suggests that exposure to adverse environmental factors (e.g., poor diet, stress, or infection) early in life can increase the likelihood of chronic diseases in adulthood. Recent reports found that transient hypoxemia during the perinatal period can increase pulmonary vasoconstriction during hypoxia exposure in adulthood [30]. Previous work from our laboratory showed that both IUGR and EUGR led to an exacerbated response to hypoxia and the development of PH in adult rats [1–3]. The results from this investigation are in agreement with the findings from previous studies, and therefore support the premise that pulmonary vascular disease in adulthood is closely related to environmental factors in early life.

### Postnatal exposure to hypoxia alters pulmonary vascular function

PH is a progressive disease consisting of multi-factorial pathogeny, which results in right heart failure with poor prognosis [31]. It is well established that there is an interaction between hypoxia and PH, thus underscoring the importance of understanding the complex interactions between hypoxia and the development of pulmonary diseases. NO signaling pathways play a key role in hypoxic PH [32]. In animal models of chronic hypoxia in adulthood, the expression of eNOS protein increases after hypoxia. However, the expression of eNOS in neonatal rats after hypoxia is still unclear. Chicoine et al. found that chronic hypoxia in neonatal rats led to a decreased expression of eNOS protein [33]. Whereas Sheak et al. showed that the expression of eNOS protein in neonatal rats did not change after hypoxia, but the expression of p-eNOS increased [34]. In the present investigation, eNOS increased following postnatal exposure to hypoxia but decreased in the adult rat. This change could suggest that eNOS expression increased when PVECs were exposed to hypoxia. However, as PH progressed, PVECs became dysfunctional even under a normoxic state and they produced less eNOS. Regulated by a variety of molecules and pathways such as VEGF, the dysfunction of PVECs plays a key role in the development of PH [35]. VEGF promotes angiogenesis and the proliferation, migration, and differentiation of endothelial cells by binding to the VEGF receptor 2 (VEGFR2) on the pulmonary vascular endothelium [34]. Consistent with previous investigations [36], our results showed that the expression of VEGF in the lung tissue is consistent with the levels of p-eNOS in both 2- and 9-week-old rats. VEGF can also affect eNOS phosphorylation, which likely alters local NO bioavailability and vasodilator signaling. These findings indicate that the mutual regulation between VEGF and p-eNOS is involved in the regulation of adult pulmonary vascular function following postnatal hypoxia.

### Potential role of m^6^A in the development of hypoxia-induced PH

Compelling evidence suggests that epigenetic modification plays an essential role in the development of PH [37]. In our previous study, we discovered that the binding of histone acetylation and hypoxia-inducible factor-1α (HIF-1α) to the endothelin-1 (ET-1) gene promoter increased in PAH following IUGR [3]. We also demonstrated the epigenetic regulation of Notch1 in the pulmonary microvascular rarefaction following EUGR [2]. Another study found that nitric oxide synthase (NOS) upregulation was associated with increased H3 and H4 histone acetylation in the eNOS promoter in a neonatal rodent persistent PH of the newborn (PPHN) model [25]. However, the epigenetic regulation of these factors in mammals and whether m^6^A mediates PH remains unknown. Since m^6^A is a common chemical modification of RNAs in mammals, it functions in various vital biological pathways such as tumorigenesis [5, 38] and embryonic [13, 39] and neuronal development [21, 40]. Considerable attention has been given to the essential role of m^6^A in embryonic development and spermatogenesis [15]. Still, there is little focus on the function of m^6^A in the lung or pulmonary vascular disease development.

We hypothesized that m^6^A also participates in PH following postnatal exposure to hypoxia. m^6^A methyltransferase and demethylase proteins exhibited lower levels in the postnatal hypoxia group than in the controls, while the total level of m^6^A in lung tissue was not affected by postnatal hypoxia. Among m^6^A-related proteins, WTAP levels did not change. This might be related to the fact that WTAP has no methyltransferase activity. Instead, it regulates m^6^A levels by combining with METTL3/METTL14 complex [41]. Future investigations should seek to identify other m^6^A-related proteins, which may help identify the specific role that m^6^A plays in the pathogenesis of hypoxia-induced PH.

METTL3 expression was consistently lower in the hypoxia group than in the controls. METTL3 participates in the development of tumors and the development of early embryos by regulating m^6^A modification [42–44]. In a recent investigation [45], METTL3 expression was significantly upregulated in patients with lung adenocarcinoma. Cytological experiments have demonstrated that METTL3 affects the growth, survival, and invasion of human lung cancer cells [45], which implies that METTL3 plays an important role in the long-term effects on pulmonary vasculature function following postnatal exposure to hypoxia.

The present investigation utilized MeRIP to analyze the specific m^6^A methylation changes following exposure to hypoxia. We found 21 hyper-methylated and 5 hypo-methylated peaks in 2-week-old rats exposed to hypoxia. In comparison, 7 peaks were hyper-methylated, and 13 peaks were hypo-methylated in adult rats who had suffered from postnatal hypoxia. While these might not seem like a significant finding, these differential peak-related genes are involved in many respiratory-related physiological processes such as respiratory tube development, Notch signal pathways, and the activation of endothelial cells. Thus, it is likely that m^6^A participates in the pathogenesis of PH. Interestingly, we found that the tricho-rhino-phalangeal syndrome 1 (*Trps1*) gene was hypomethylated in both the 2-week-old and 9-week-old rats who were exposed to postnatal hypoxia. TRPS1, a member of the GATA transcription factor family, is widely expressed in many tissues and organs and plays a critical role in mammalian development and differentiation [46, 47]. TRPS1 is also highly expressed in lung cancer [48] and is involved in regulating epithelial-to-mesenchymal transition (EMT) during embryonic development [49]. While EMT plays an important role in the occurrence and development of PH [50], it has not been reported whether *Trps1* is also involved in the regulation of PH pathogenesis. Our results suggest that *Trps1* mRNA methylation can regulate PH following postnatal hypoxia by affecting EMT in the newborn rat, and this effect can persist into adulthood. Future investigations will identify the specific role(s) of EMT in the development of hypoxia-induced PH.

### Experimental considerations and future directions

While this investigation demonstrated that postnatal exposure to hypoxia impacts pulmonary vascular function and development, it is unclear if this results in impaired functional capacity (i.e., exercise tolerance) of these animals. Considering that PH leads to reduced maximal oxygen uptake and exercise capacity in humans [51], it is likely that the impaired pulmonary vascular function in rats exposed to postnatal hypoxia studied herein would have reduced exercise capacity. Future investigations into the interplay between postnatal hypoxia-induced PH and reduced exercise tolerance may aid in the bench-to-bedside translation of our understanding of this disease and the associated molecular mechanisms.

This investigation sheds light on the long-term effect of postnatal exposure to hypoxia on pulmonary vascular functions and the role of m^6^A in regulating PH. These results suggest that m^6^A methylation is a biomarker of epigenetic modification of critical genes that regulate pulmonary arterial pressure and lung development and, therefore, may be potential therapeutic targets. However, additional investigations are required to delineate the specific regulatory mechanisms between m^6^A and PH and test the possibilities of regulating m^6^A methylation to treat PH.

## Conclusions

To summarize, our results suggest that postnatal hypoxia can cause PH, which can persist into adulthood. The development of PH may be due to the continuous low expression of METTL3, which impacts the m^6^A level of PH-related genes. These findings offer a new perspective to explain the molecular mechanisms of lung diseases and provide a reference for future investigations into therapeutic development.

## Data Availability

The datasets used and analyzed during the current study are available from the corresponding author on reasonable request.

## Abbreviations

ALKBH5: A-ketoglutarate-dependent dioxygenase alkB homolog 5
DEGs: Differentially expressed genes
DOHaD: Developmental origins of health and disease
EMT: Epithelial-to-mesenchymal transition
eNOS: Endothelial nitric oxide synthase
ET-1: Endothelin-1
EUGR: Extrauterine growth retardation
Ttll12: Tubulin tyrosine ligase like 12
FTO: Fat mass- and obesity-associated protein
Gli3: GLI family zinc finger 3
GO analysis: Gene Ontology analysis
HE: Hematoxylin–eosin staining
HIF-1α: Hypoxia-inducible factor-1α
IHC: Immunohistochemical staining
IUGR: Intrauterine growth retardation
KEGG analysis: Kyoto Encyclopedia of Genes and Genomes pathway enrichment analysis
m^6^A: N^6^-methyladenosine
MACS: Magnetic activated cell sorting
MAN: Mean alveolar number
MeRIP-seq: Methylated RNA immunoprecipitation sequencing
METTL14: Methyltransferase like 14
METTL3: Methyltransferase like 3
mPAP: Mean pulmonary arterial pressure
Nog: Noggin
NOS: Nitric oxide synthase
PAH: Pulmonary arterial hypertension
Pbm: Perimeter of pulmonary vascular basement membrane
PE-50: A polyethylene catheter
p-eNOS: Phosphorylated endothelial nitric oxide synthase
PH: Pulmonary hypertension
PPHN: Persistent pulmonary hypertension of the newborn
PVECs: Pulmonary vascular endothelial cells
qPCR: Quantitative real-time PCR
Rras2: RAS related 2
RVP: Right ventricular pressure
Tc1: Transcriptional and immune response regulator
Trps1: Tricho-rhino-phalangeal syndrome 1
Vangl2: VANGL planar cell polarity protein 2
VEGF: Vascular endothelial growth factor
VEGFR2: Vascular endothelial growth factor receptor 2
WTAP: Wilms’ tumor 1-associating protein
YTH family: YTH domain-containing family
α-SMA: Smooth muscle specific alpha-actin antibody

## References

[1] Zhang L, Tang L, Wei J, Lao L, Gu W, Hu Q, Lv Y, Fu L, Du L (2014). Extrauterine growth restriction on pulmonary vascular endothelial dysfunction in adult male rats: the role of epigenetic mechanisms. J Hypertens.

[2] Tang LL, Zhang LY, Lao LJ, Hu QY, Gu WZ, Fu LC, Du LZ (2015). Epigenetics of Notch1 regulation in pulmonary microvascular rarefaction following extrauterine growth restriction. Respir Res.

[3] Xu X-F, Lv Y, Gu W-Z, Tang L-L, We J-K, Zhang L-Y, Du L-Z (2013). Epigenetics of hypoxic pulmonary arterial hypertension following intrauterine growth retardation rat epigenetics in PAH following IUGR. Respir Res.

[4] Panneerdoss S, Eedunuri VK, Yadav P, Timilsina S, Rajamanickam S, Viswanadhapalli S, Abdelfattah N, Onyeagucha BC, Cui X, Lai Z, Mohammad TA, Gupta YK, Huang THM, Huang Y, Chen Y, Rao MK (2018). Cross-talk among writers, readers, and erasers of m6A regulates cancer growth and progression. Sci Adv..

[5] Hong K (2018). Emerging function of N6-methyladenosine in cancer (Review). Oncol Lett.

[6] Desrosiers R, Friderici K, Rottman F (1974). Identification of methylated nucleosides in messenger RNA from Novikoff hepatoma cells. Proc Natl Acad Sci U S A.

[7] Niu Y, Zhao X, Wu YS, Li MM, Wang XJ, Yang YG (2013). N6-methyl-adenosine (m6A) in RNA: an old modification with a novel epigenetic function. Genomics Proteom Bioinform.

[8] Yue Y, Liu J, He C (2015). RNA N6-methyladenosine methylation in post-transcriptional gene expression regulation. Genes Dev.

[9] Wang X, Lu Z, Gomez A, Hon GC, Yue Y, Han D, Fu Y, Parisien M, Dai Q, Jia G, Ren B, Pan T, He C (2014). N6-methyladenosine-dependent regulation of messenger RNA stability. Nature.

[10] Wang X, Zhao BS, Roundtree IA, Lu Z, Han D, Ma H, Weng X, Chen K, Shi H, He C (2015). N(6)-methyladenosine modulates messenger RNA translation efficiency. Cell.

[11] Roundtree IA, Luo GZ, Zhang Z, Wang X, Zhou T, Cui Y, Sha J, Huang X, Guerrero L, Xie P, He E, Shen B, He C. YTHDC1 mediates nuclear export of N(6)-methyladenosine methylated mRNAs. eLife. 2017;6: 31311. 10.7554/eLife.31311 PMC5648532 28984244

[12] Shi H, Wang X, Lu Z, Zhao BS, Ma H, Hsu PJ, Liu C, He C (2017). YTHDF3 facilitates translation and decay of N(6)-methyladenosine-modified RNA. Cell Res.

[13] Batista PJ, Molinie B, Wang J, Qu K, Zhang J, Li L, Bouley DM, Lujan E, Haddad B, Daneshvar K, Carter AC, Flynn RA, Zhou C, Lim KS, Dedon P, Wernig M, Mullen AC, Xing Y, Giallourakis CC, Chang HY (2014). m(6)A RNA modification controls cell fate transition in mammalian embryonic stem cells. Cell Stem Cell.

[14] Fustin JM, Doi M, Yamaguchi Y, Hida H, Nishimura S, Yoshida M, Isagawa T, Morioka MS, Kakeya H, Manabe I, Okamura H (2013). RNA-methylation-dependent RNA processing controls the speed of the circadian clock. Cell.

[15] Lin Z, Hsu PJ, Xing X, Fang J, Lu Z, Zou Q, Zhang KJ, Zhang X, Zhou Y, Zhang T, Zhang Y, Song W, Jia G, Yang X, He C, Tong MH (2017). Mettl3-/Mettl14-mediated mRNA N(6)-methyladenosine modulates murine spermatogenesis. Cell Res.

[16] Engel M, Eggert C, Kaplick PM, Eder M, Roh S, Tietze L, Namendorf C, Arloth J, Weber P, Rex-Haffner M, Geula S, Jakovcevski M, Hanna JH, Leshkowitz D, Uhr M, Wotjak CT, Schmidt MV, Deussing JM, Binder EB, Chen A (2018). The role of m(6)A/m-RNA methylation in stress response regulation. Neuron.

[17] Chang M, Lv H, Zhang W, Ma C, He X, Zhao S, Zhang ZW, Zeng YX, Song S, Niu Y, Tong WM (2017). Region-specific RNA m(6)A methylation represents a new layer of control in the gene regulatory network in the mouse brain. Open Biol.

[18] Wang CX, Cui GS, Liu X, Xu K, Wang M, Zhang XX, Jiang LY, Li A, Yang Y, Lai WY, Sun BF, Jiang GB, Wang HL, Tong WM, Li W, Wang XJ, Yang YG, Zhou Q (2018). METTL3-mediated m6A modification is required for cerebellar development. PLoS Biol.

[19] Zhao BS, Wang X, Beadell AV, Lu Z, Shi H, Kuuspalu A, Ho RK, He C (2017). m(6)A-dependent maternal mRNA clearance facilitates zebrafish maternal-to-zygotic transition. Nature.

[20] Meyer KD, Saletore Y, Zumbo P, Elemento O, Mason CE, Jaffrey SR (2012). Comprehensive analysis of mRNA methylation reveals enrichment in 3' UTRs and near stop codons. Cell.

[21] Ma C, Chang M, Lv H, Zhang ZW, Zhang W, He X, Wu G, Zhao S, Zhang Y, Wang D, Teng X, Liu C, Li Q, Klungland A, Niu Y, Song S, Tong WM (2018). RNA m(6)A methylation participates in regulation of postnatal development of the mouse cerebellum. Genome Biol.

[22] Ito K, Ito M, Elliott WM, Cosio B, Caramori G, Kon OM, Barczyk A, Hayashi S, Adcock IM, Hogg JC, Barnes PJ (2005). Decreased histone deacetylase activity in chronic obstructive pulmonary disease. N Engl J Med.

[23] Cosio BG, Mann B, Ito K, Jazrawi E, Barnes PJ, Chung KF, Adcock IM (2004). Histone acetylase and deacetylase activity in alveolar macrophages and blood mononocytes in asthma. Am J Respir Crit Care Med.

[24] Saco TV, Parthasarathy PT, Cho Y, Lockey RF, Kolliputi N (2014). Role of epigenetics in pulmonary hypertension. Am J Physiol Cell Physiol.

[25] Xu XF, Ma XL, Shen Z, Wu XL, Cheng F, Du LZ (2010). Epigenetic regulation of the endothelial nitric oxide synthase gene in persistent pulmonary hypertension of the newborn rat. J Hypertens.

[26] Wu X, Du L, Xu X, Tan L, Li R (2010). Increased nitrosoglutathione reductase activity in hypoxic pulmonary hypertension in mice. J Pharmacol Sci.

[27] Dai J, Ji B, Zhao G, Lu Y, Liu Y, Mou Q, Sakurai R, Xie Y, Zhang Q, Xu S, Rehan VK (2020). Developmental timing determines the protective effect of maternal electroacupuncture on perinatal nicotine exposure-induced offspring lung phenotype. Biomed Res Int.

[28] Zhang Z, Luo X, Lv Y, Yan L, Xu S, Wang Y, Zhong Y, Hang C, Jyotsnav J, Lai D, Shen Z, Xu X, Ma X, Chen Z, Pan Y, Du L (2019). Intrauterine growth restriction programs intergenerational transmission of pulmonary arterial hypertension and endothelial dysfunction via sperm epigenetic modifications. Hypertension.

[29] Dominissini D, Moshitch-Moshkovitz S, Salmon-Divon M, Amariglio N, Rechavi G (2013). Transcriptome-wide mapping of N(6)-methyladenosine by m(6)A-seq based on immunocapturing and massively parallel sequencing. Nat Protoc.

[30] O'Callaghan DS, Savale L, Montani D, Jais X, Sitbon O, Simonneau G, Humbert M (2011). Treatment of pulmonary arterial hypertension with targeted therapies. Nat Rev Cardiol.

[31] Ranchoux B, Harvey LD, Ayon RJ, Babicheva A, Bonnet S, Chan SY, Yuan JX, Perez VJ (2018). Endothelial dysfunction in pulmonary arterial hypertension: an evolving landscape (2017 Grover Conference Series). Pulm Circ.

[32] He Q, Liu X, Zhong Y, Xu SS, Zhang ZM, Tang LL, Zhang LY, Du LZ (2018). Arginine bioavailability and endothelin-1 system in the regulation of vascular function of umbilical vein endothelial cells from intrauterine growth restricted newborns. Nutr Metab Cardiovasc Dis.

[33] Chicoine LG, Avitia JW, Deen C, Nelin LD, Earley S, Walker BR (2002). Developmental differences in pulmonary eNOS expression in response to chronic hypoxia in the rat. J Appl Physiol.

[34] Sheak JR, Weise-Cross L, deKay RJ, Walker BR, Jernigan NL, Resta TC (2017). Enhanced NO-dependent pulmonary vasodilation limits increased vasoconstrictor sensitivity in neonatal chronic hypoxia. Am J Physiol Heart Circ Physiol.

[35] Mortola JP, Xu L, Lauzon A-M (1990). Body growth, lung and heart weight, and DNA content in newborn rats exposed to different levels of chronic hypoxia. Can J Physiol Pharmacol..

[36] Liu Y, Paterson M, Baumgardt SL, Irwin MG, Xia Z, Bosnjak ZJ, Ge ZD (2019). Vascular endothelial growth factor regulation of endothelial nitric oxide synthase phosphorylation is involved in isoflurane cardiac preconditioning. Cardiovasc Res.

[37] Kim GH, Ryan JJ, Marsboom G, Archer SL (2011). Epigenetic mechanisms of pulmonary hypertension. Pulm Circ.

[38] Li Z, Weng H, Su R, Weng X, Zuo Z, Li C, Huang H, Nachtergaele S, Dong L, Hu C, Qin X, Tang L, Wang Y, Hong GM, Huang H, Wang X, Chen P, Gurbuxani S, Arnovitz S, Li Y, Li S, Strong J, Neilly MB, Larson RA, Jiang X, Zhang P, Jin J, He C, Chen J (2017). FTO plays an oncogenic role in acute myeloid leukemia as a N(6)-methyladenosine RNA demethylase. Cancer Cell.

[39] Mendel M, Chen KM, Homolka D, Gos P, Pandey RR, McCarthy AA, Pillai RS (2018). Methylation of structured RNA by the m(6)A writer METTL16 is essential for mouse embryonic development. Mol Cell.

[40] Lence T, Akhtar J, Bayer M, Schmid K, Spindler L, Ho CH, Kreim N, Andrade-Navarro MA, Poeck B, Helm M, Roignant JY (2016). m(6)A modulates neuronal functions and sex determination in Drosophila. Nature.

[41] Liu J, Yue Y, Han D, Wang X, Fu Y, Zhang L, Jia G, Yu M, Lu Z, Deng X, Dai Q, Chen W, He C (2014). A METTL3-METTL14 complex mediates mammalian nuclear RNA N6-adenosine methylation. Nat Chem Biol.

[42] Wu Y, Xie L, Wang M, Xiong Q, Guo Y, Liang Y, Li J, Sheng R, Deng P, Wang Y, Zheng R, Jiang Y, Ye L, Chen Q, Zhou X, Lin S, Yuan Q (2018). Mettl3-mediated m(6)A RNA methylation regulates the fate of bone marrow mesenchymal stem cells and osteoporosis. Nat Commun.

[43] Chen M, Wei L, Law CT, Tsang FH, Shen J, Cheng CL, Tsang LH, Ho DW, Chiu DK, Lee JM, Wong CC, Ng IO, Wong CM (2018). RNA N6-methyladenosine methyltransferase-like 3 promotes liver cancer progression through YTHDF2-dependent posttranscriptional silencing of SOCS2. Hepatology.

[44] Barbieri I, Tzelepis K, Pandolfini L, Shi J, Millan-Zambrano G, Robson SC, Aspris D, Migliori V, Bannister AJ, Han N, De Braekeleer E, Ponstingl H, Hendrick A, Vakoc CR, Vassiliou GS, Kouzarides T (2017). Promoter-bound METTL3 maintains myeloid leukaemia by m(6)A-dependent translation control. Nature.

[45] Lin S, Choe J, Du P, Triboulet R, Gregory RI (2016). The m(6)A methyltransferase METTL3 promotes translation in human cancer cells. Mol Cell.

[46] Malik TH, Shoichet SA, Latham P, Kroll TG, Shivdasani RA (2001). Transcriptional repression and developmental functions of the atypical vertebrate GATA protein TRPS1. EMBO J..

[47] Suemoto H, Muragaki Y, Nishioka K, Sato M, Ooshima A, Itoh S, Hatamura I, Ozaki M, Braun A, Gustafsson E, Fassler R (2007). Trps1 regulates proliferation and apoptosis of chondrocytes through Stat3 signaling. Dev Biol.

[48] Liu H, Liao Y, Tang M, Wu T, Tan D, Zhang S, Wang H (2018). Trps1 is associated with the multidrug resistance of lung cancer cell by regulating MGMT gene expression. Cancer Med.

[49] Su P, Hu J, Zhang H, Jia M, Li W, Jing X, Zhou G (2014). Association of TRPS1 gene with different EMT markers in ERα-positive and ERα-negative breast cancer. Diagn Pathol..

[50] Ranchoux B, Antigny F, Rucker-Martin C, Hautefort A, Pechoux C, Bogaard HJ, Dorfmuller P, Remy S, Lecerf F, Plante S, Chat S, Fadel E, Houssaini A, Anegon I, Adnot S, Simonneau G, Humbert M, Cohen-Kaminsky S, Perros F (2015). Endothelial-to-mesenchymal transition in pulmonary hypertension. Circulation.

[51] Gläser S, Noga O, Koch B, Opitz CF, Schmidt B, Temmesfeld B, Dörr M, Ewert R, Schäper C (2009). Impact of pulmonary hypertension on gas exchange and exercise capacity in patients with pulmonary fibrosis. Respir Med.

